# Supplementary material for: Decoding molecular programs in melanoma brain metastases
Source: Nat Commun. 2022 Nov 26;13:7304. doi: 10.1038/s41467-022-34899-x (PMC9701224; doi:10.1038/s41467-022-34899-x)
Supplement: Supplementary file 1 — Supplementary Information [file 41467_2022_34899_MOESM1_ESM.pdf]

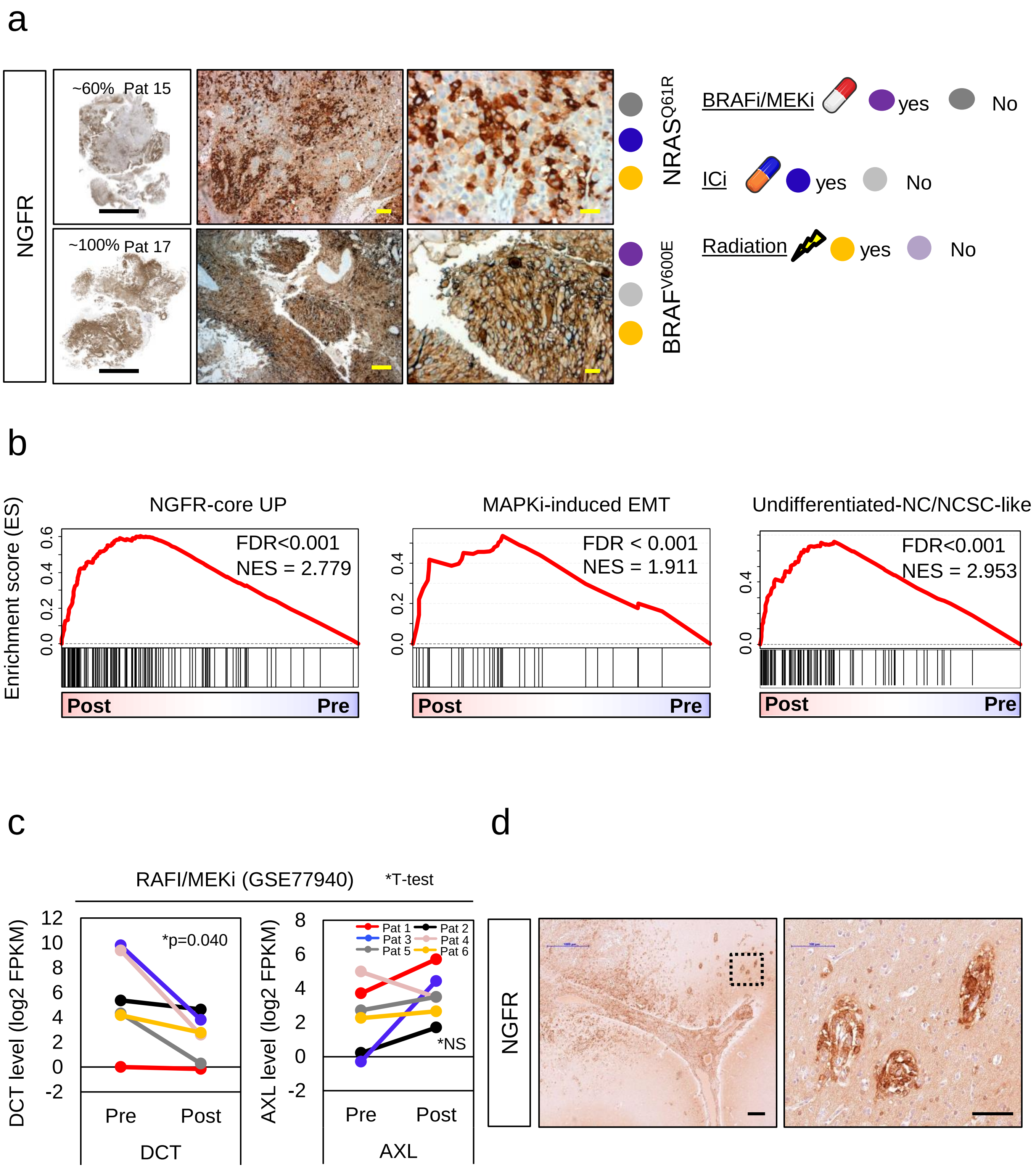
a
NGFR
~60% Pat 15
~100% Pat 17
NRAS^{Q61R}
BRAF^{V600E}
BRAFi/MEKi yes No
ICi yes No
Radiation yes No
b
NGFR-core UP
FDR<0.001
NES = 2.779
MAPKi-induced EMT
FDR < 0.001
NES = 1.911
Undifferentiated-NC/NCSC-like
FDR<0.001
NES = 2.953
Enrichment score (ES)
Post
Pre
c
RAFI/MEKi (GSE77940)
*T-test
*p=0.040
DCT level (log2 FPKM)
Pre
Post
DCT
Pat 1
Pat 2
Pat 3
Pat 4
Pat 5
Pat 6
*NS
AXL level (log2 FPKM)
AXL
d
NGFR

Supplementary fig. 1: Therapeutic interventions affect phenotypic states of tumor cells. a.) Immunohistochemistry (IHC) for NGFR of two representative MBM of patients (Pt. 15, 17) who received combinatory therapies: BRAFi/MEKi (dabrafenib/trametinib), ICi (nivolumab and/or ipilimumab) or radiation therapy. Scale bars indicate 50 µm. Left: whole slide scans, scale bars indicate 1,000 µm. b.) GSEA of patient-matched melanoma (n=12): pre- (n=6) and post-BRAFi/MEKi (n=6) therapy (study GSE77940) indicating enrichment of NGFR-driven networks, MAPK-inhibitor induced EMT or undifferentiated, neural crest (NC)-like phenotypes. c.) Investigation of expression levels of DCT and AXL in samples analyzed in (b). Significance was determined by a paired two-tailed t-test, n=6 tumors per group were investigated. d.) IHC of a highly invasive MBM of a patient who was refractory to BRAFi/MEKi, ICi and radiation therapy for expression of NGFR indicating the presence of several $NGFR^+$ micrometastases that infiltrated BTE. Scale bars indicate 100 µm.

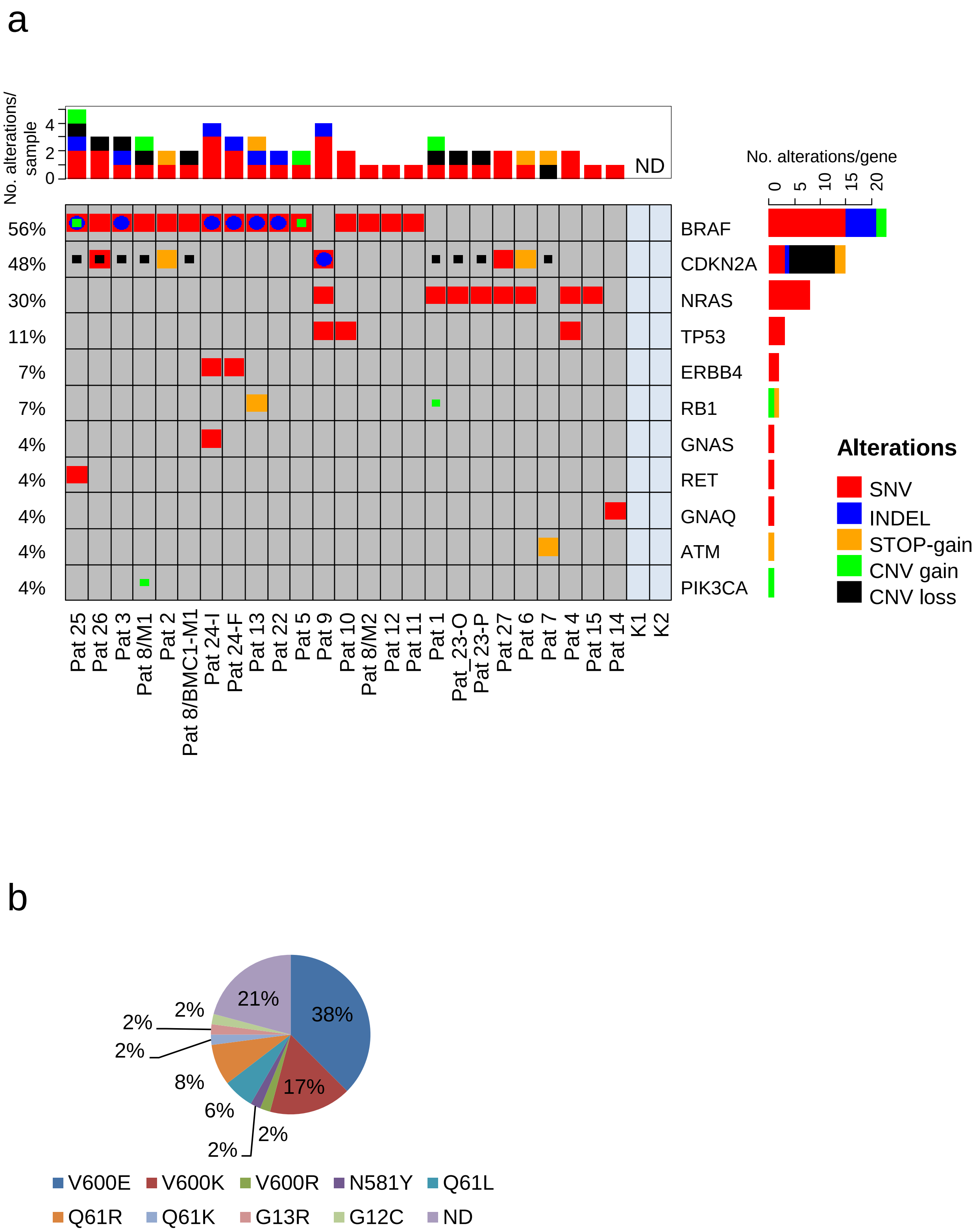

a
No. alterations/sample
0
2
4
ND
No. alterations/gene
0
5
10
15
20
56%
48%
30%
11%
7%
7%
4%
4%
4%
4%
4%
BRAF
CDKN2A
NRAS
TP53
ERBB4
RB1
GNAS
RET
GNAQ
ATM
PIK3CA
Alterations
SNV
INDEL
STOP-gain
CNV gain
CNV loss
Pat 25
Pat 26
Pat 3
Pat 8/M1
Pat 2
Pat 8/BMC1-M1
Pat 24-I
Pat 24-F
Pat 13
Pat 22
Pat 5
Pat 9
Pat 10
Pat 8/M2
Pat 12
Pat 11
Pat 1
Pat_23-O
Pat 23-P
Pat 27
Pat 6
Pat 7
Pat 4
Pat 15
Pat 14
K1
K2
b
38%
17%
21%
2%
2%
2%
8%
6%
2%
2%
V600E
V600K
V600R
N581Y
Q61L
Q61R
Q61K
G13R
G12C
ND

Supplementary figure 2: Genetic characteristics of MBM. a.) TargetSeq of MBM and BC (K1, K2) using a 50 gene panel (CHP2v) revealed mutations (single nucleotide variants, SNV) in 11 genes and provided insight into copy number changes (CNV gain, loss), insertion and deletions (INDEL) and nonsense mutations (STOP-gain). b.) Types of BRAF and NRAS mutations identified in (a), the mutation status was not determined (ND) in 10 MBM (21%).

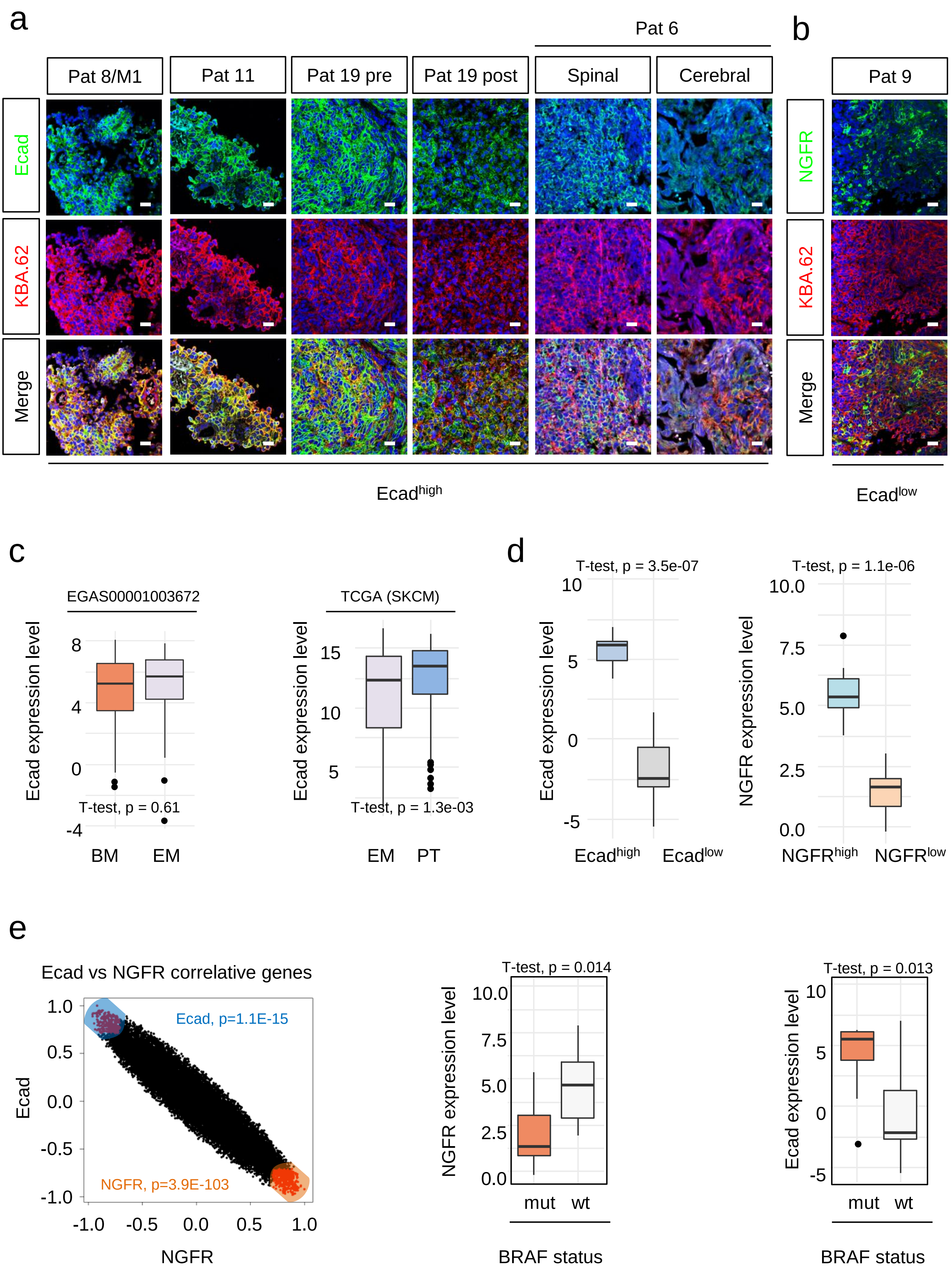
a
Pat 6
Pat 8/M1
Pat 11
Pat 19 pre
Pat 19 post
Spinal
Cerebral
Ecad
KBA.62
Merge
Ecad^high
b
Pat 9
NGFR
KBA.62
Merge
Ecad^low
c
EGAS00001003672
Ecad expression level
8
4
0
-4
T-test, p = 0.61
BM
EM
TCGA (SKCM)
Ecad expression level
15
10
5
T-test, p = 1.3e-03
EM
PT
d
T-test, p = 3.5e-07
Ecad expression level
10
5
0
-5
Ecad^high
Ecad^low
T-test, p = 1.1e-06
NGFR expression level
10.0
7.5
5.0
2.5
0.0
NGFR^high
NGFR^low
e
Ecad vs NGFR correlative genes
Ecad, p=1.1E-15
NGFR, p=3.9E-103
Ecad
1.0
0.5
0.0
-0.5
-1.0
-1.0
-0.5
0.0
0.5
1.0
NGFR
T-test, p = 0.014
NGFR expression level
10.0
7.5
5.0
2.5
0.0
mut
wt
BRAF status
T-test, p = 0.013
Ecad expression level
10
5
0
-5
mut
wt
BRAF status

Supplementary fig. 3: Ecad and NGFR expression define molecular subgroups of MBM. a.) IF and confocal microscopy of epithelial-like MBM (Pt.8/M1, 11), matched pre- (Pat19 pre, frontal lobe, left) and post-relapse (Pat19 post, cerebellum, left) tumors and extracranial (spinal) and cerebral metastases of Pat6 revealed presence of KBA.62 and intense staining and proper membrane localization of Ecad (~80% of cells). In Pat19 post exhibited a reduced level (~50%) and punctuated localization of Ecad but comparable levels in spinal and concordant cerebral metastases. Bars indicate 50 µm. b.) Expression of NGFR in a representative Ecad^{low} tumor (Pat 9). c.) Left panel: Box plots depicting levels of Ecad expression in an independent set of MBM (n=79) and EM (n=59), study EGAS00001003672. Statistical testing revealed no significant difference (p=0.61) of Ecad levels. (c). Right panel: Ecad expression in EM (n=321) and primary tumors (PT; n=151) of the TCGA-SKCM data set. d.) Box plots depict that levels of Ecad or *NGFR* significantly subdivide MBM (n=16) into subgroups, (Ecad^{high} vs. low, p=3.5e-07; NGFR^{high} vs. low, p=1.1.e-06), right panels. e.) Left panel: Comparison of Ecad and *NGFR* correlated genes revealed a mutually exclusive pattern. Center and right panels: Box plots depicting levels of *NGFR* and Ecad in BRAF^{mut} and BRAF^{wt} tumors (n=16). In c, d, e box and whisker plots show median (center line), the upper and lower quartiles (the box), and the range of the data (the whiskers), including outliers and significance was determined by a unpaired two-tailed, t-test. Source data are provided as a Source Data file.

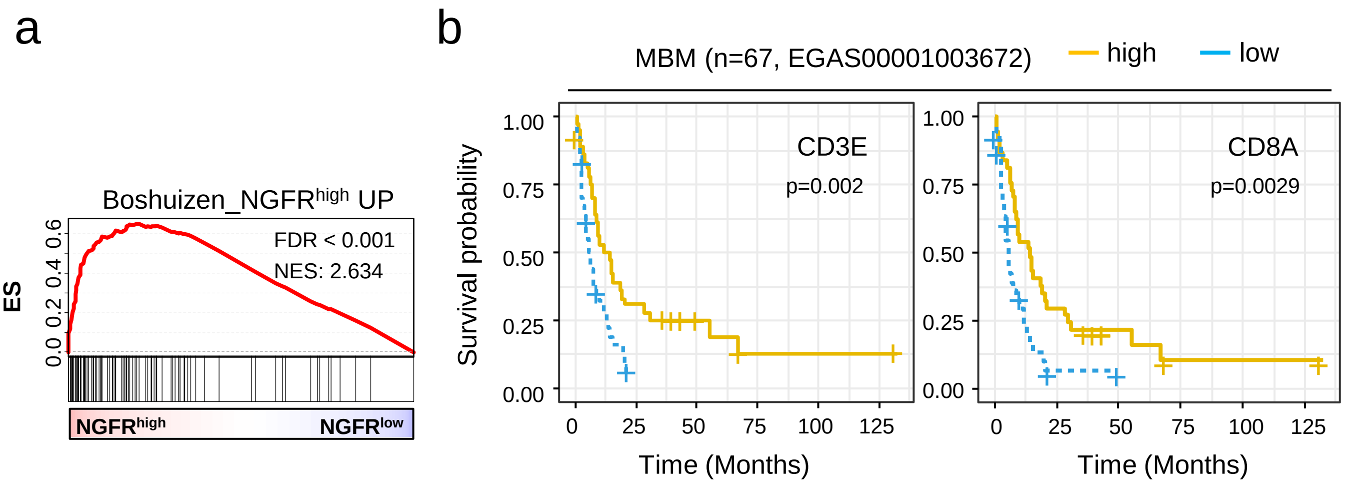
a
b
Boshuizen_NGFRhigh UP
FDR < 0.001
NES: 2.634
ES
0.0 0.2 0.4 0.6
NGFRhigh
NGFRlow
MBM (n=67, EGAS00001003672)
high
low
CD3E
p=0.002
CD8A
p=0.0029
Survival probability
1.00
0.75
0.50
0.25
0.00
0
25
50
75
100
125
Time (Months)
Time (Months)

Supplementary fig. 4: Presence of T cells is associated with favorable outcome. a.) GSEA of $NGFR^{high}$ and $NGFR^{low}$ subsets revealed enrichment of a NGFR-driven signature in $NGFR^{high}$ MBM. b.) Kaplan-Meier survival analysis demonstrating favored survival of patients with MBM (study EGAS00001003672) exhibiting high levels of infiltration of naïve (*CD3E*) or cytotoxic (*CD8A*) T cells. Significance was determined by log-rank test, p-values are not corrected for multiple testing. Source data are provided as a Source Data file.

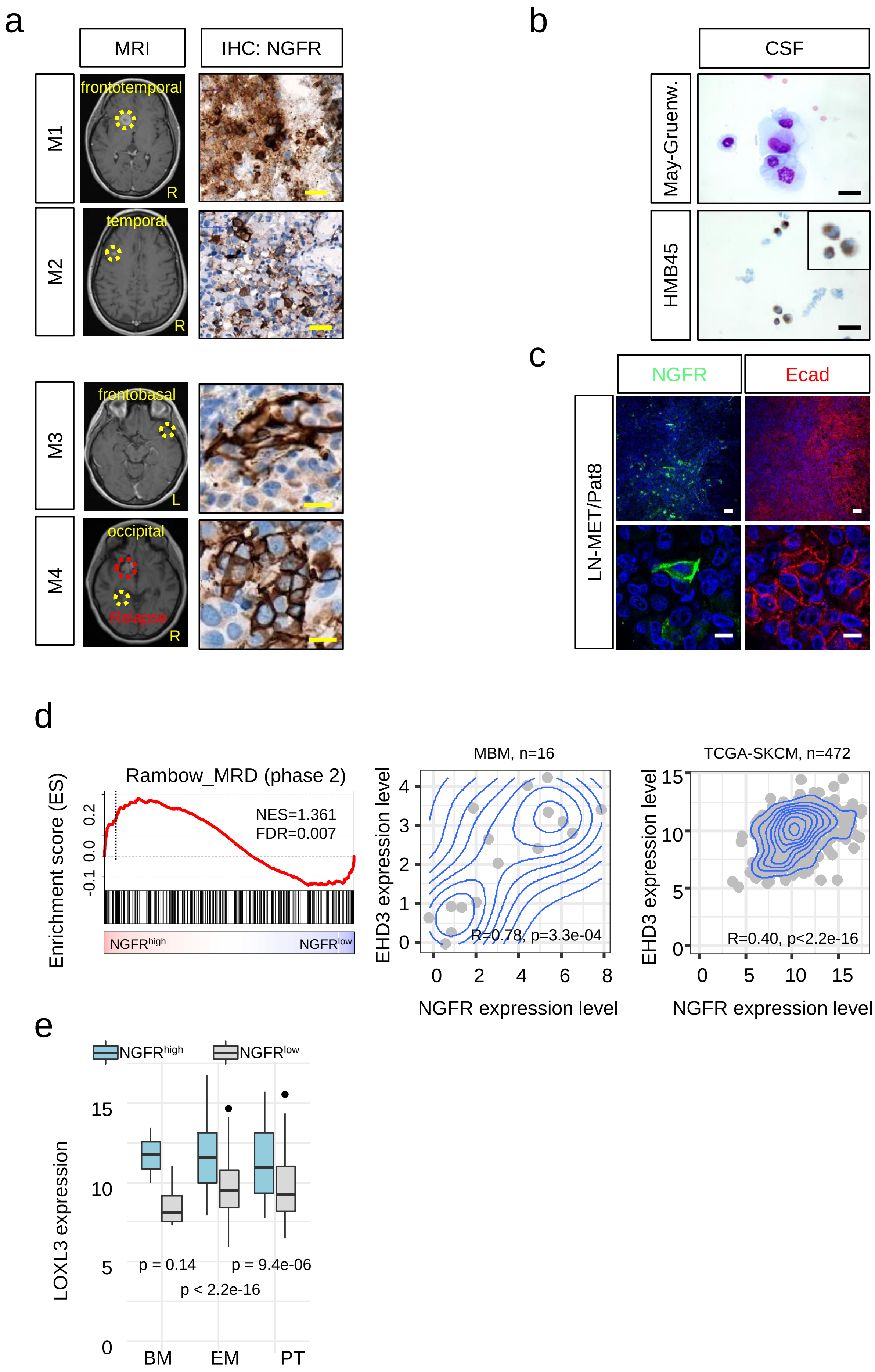

a
MRI
IHC: NGFR
M1
frontotemporal
R
M2
temporal
R
M3
frontobasal
L
M4
occipital
Relapse
R
b
CSF
May-Gruenw.
HMB45
c
NGFR
Ecad
LN-MET/Pat8
d
Rambow_MRD (phase 2)
NES=1.361
FDR=0.007
Enrichment score (ES)
NGFRhigh
NGFRlow
MBM, n=16
R=0.78, p=3.3e-04
EHD3 expression level
NGFR expression level
TCGA-SKCM, n=472
R=0.40, p<2.2e-16
EHD3 expression level
NGFR expression level
e
NGFRhigh
NGFRlow
LOXL3 expression
p = 0.14
p = 9.4e-06
p < 2.2e-16
BM
EM
PT

Supplementary fig. 5: NGFR expression discriminates MBM. a.) Left panels: Magnetic resonance imaging (MRI) of M1, M2 and M3, M4, likely reflecting MBM at different progression stages. Right panels: IHC for NGFR indicates rare $NGFR^+$ cells in M1, M2 but a high level of NGFR expression in M3, M4. b.) May-Gruenwald (May-Gruenw.) staining and IHC for HMB45 indicates the presence of MBM cells in CSF. c.) IF of a lymph node metastasis (LN-MET) of Pat8 for *NGFR* and Ecad. Bars indicate 50 µm. d.) Left panel: GSEA demonstrating enrichment of a MRD tumor cell-stage in $NGFR^{high}$ MBM. Center and right panels: Significance of Pearson correlation of NGFR and EHD3 in MBM of this study (p=3.3e-04) or TCGA-SKCM melanoma (p<2.2e-16) was determined by an unpaired two-tailed, t-test. e.) Box plot indicating significantly increased levels of *LOXL3* in extracranial metastases (EM, n=321; p<2.2e-16) and primary tumors (PT, n=151; p=9.4e-06) showing a $NGFR^{high}$ phenotype. N=472 TCGA-SKCM melanoma were investigated. Box and whisker plots show median (center line), the upper and lower quartiles (the box), and the range of the data (the whiskers), including outliers and significance was determined by a two-way anova. Source data are provided as a Source Data file.

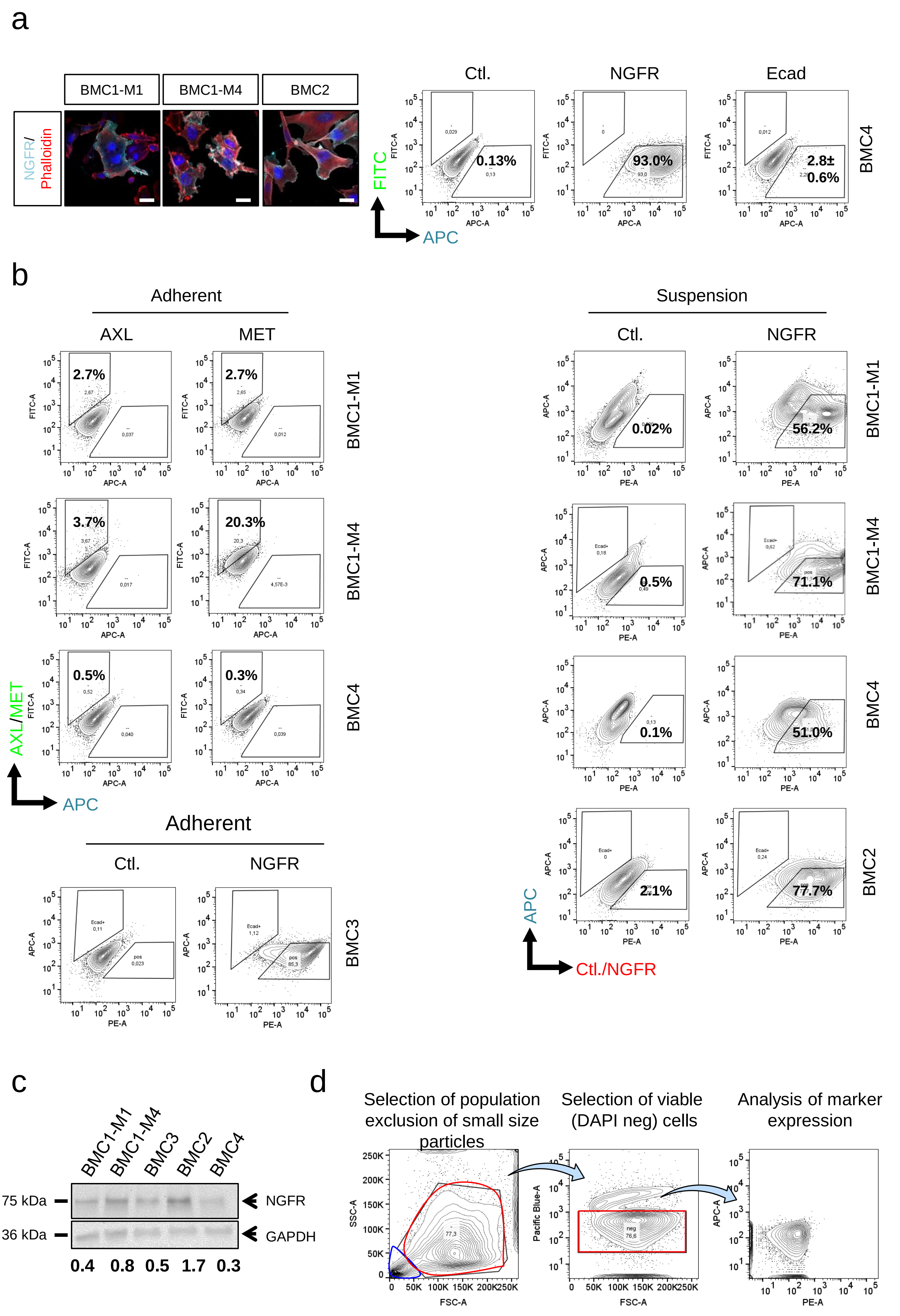

a
BMC1-M1
BMC1-M4
BMC2
NGFR/ Phalloidin
Ctl.
NGFR
Ecad
0.13%
93.0%
2.8± 0.6%
BMC4
FITC
APC
b
Adherent
Suspension
AXL
MET
Ctl.
NGFR
2.7%
2.7%
3.7%
20.3%
0.5%
0.3%
0.02%
56.2%
0.5%
71.1%
0.1%
51.0%
2.1%
77.7%
BMC1-M1
BMC1-M4
BMC4
BMC2
AXL/MET
APC
Adherent
Ctl.
NGFR
BMC3
APC
Ctl./NGFR
c
BMC1-M1
BMC1-M4
BMC3
BMC2
BMC4
75 kDa
NGFR
36 kDa
GAPDH
0.4
0.8
0.5
1.7
0.3
d
Selection of population exclusion of small size particles
Selection of viable (DAPI neg) cells
Analysis of marker expression
SSC-A
FSC-A
Pacific Blue-A
APC-A
PE-A

Supplementary fig. 6: Phenotypical characterization of BMCs. a.) Left panels: IF of a representative set of indicated BMCs for NGFR (turquoise) and phalloidin (red), bars indicate 50 µm. Center and right panels: Flow cytometric analysis of viable, adherently grown BMCs for cell surface expression of NGFR and Ecad (BMC4). b.) Left: Flow cytometry of BMCs for cell surface expression of potential drivers of migration AXL and MET (FITC-labeled), (upper panels) and NGFR (APC-labeled, lower panels). Right panels: Cell surface levels of NGFR of suspension cells (upper, lower panels). 50,000 cells were monitored. c.) Immunoblot analysis of whole cell lysates of indicated BMCs for levels of NGFR, GAPDH served as loading control. Source data are provided as a Source Data file. d.) General strategy for gating/defining cell populations in flow cytometric/ cell sorting analyses.

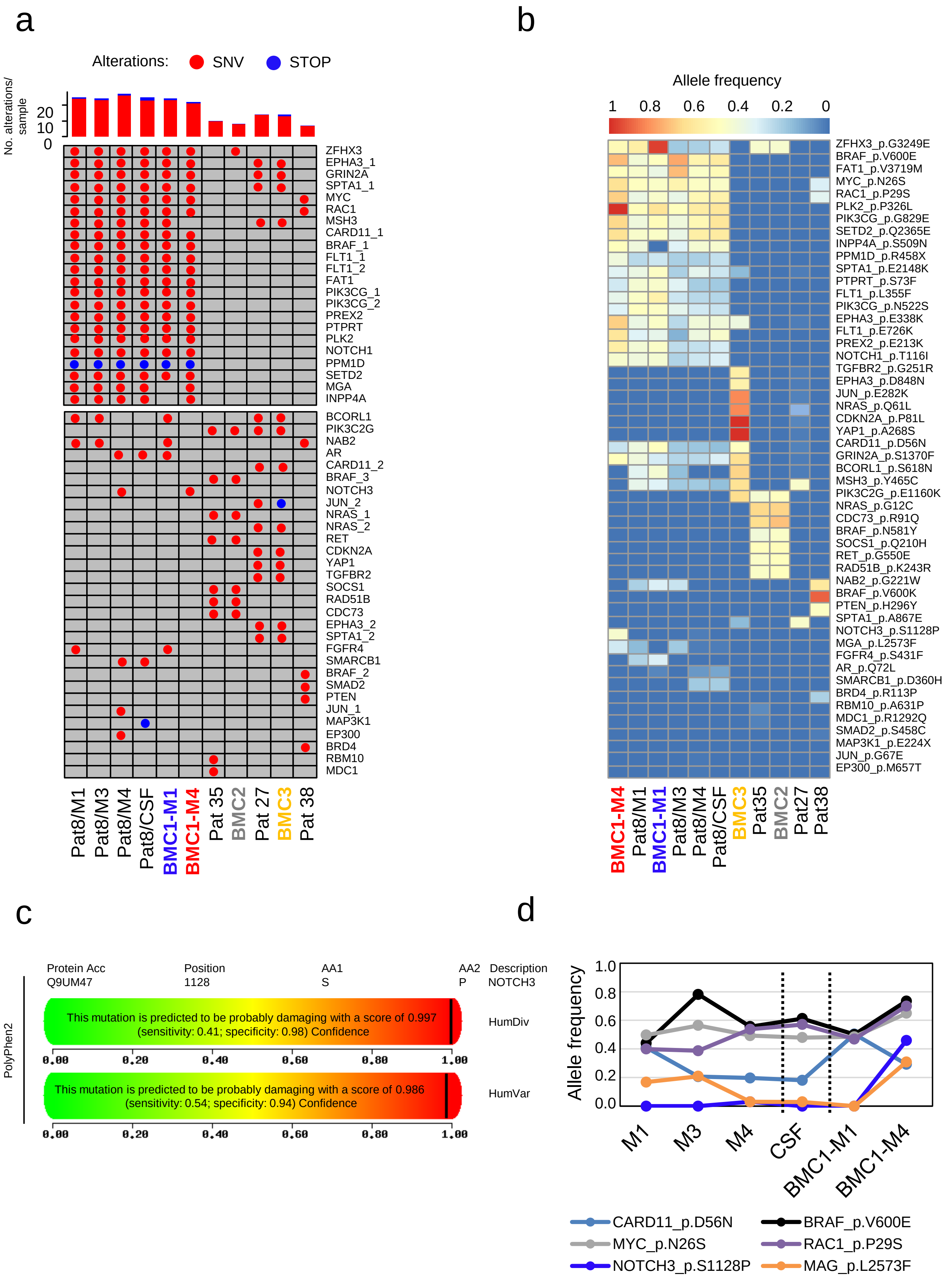

a
Alterations:
SNV
STOP
No. alterations/ sample
20
10
0
ZFHX3
EPHA3_1
GRIN2A
SPTA1_1
MYC
RAC1
MSH3
CARD11_1
BRAF_1
FLT1_1
FLT1_2
FAT1
PIK3CG_1
PIK3CG_2
PREX2
PTPRT
PLK2
NOTCH1
PPM1D
SETD2
MGA
INPP4A
BCORL1
PIK3C2G
NAB2
AR
CARD11_2
BRAF_3
NOTCH3
JUN_2
NRAS_1
NRAS_2
RET
CDKN2A
YAP1
TGFBR2
SOCS1
RAD51B
CDC73
EPHA3_2
SPTA1_2
FGFR4
SMARCB1
BRAF_2
SMAD2
PTEN
JUN_1
MAP3K1
EP300
BRD4
RBM10
MDC1
Pat8/M1
Pat8/M3
Pat8/M4
Pat8/CSF
BMC1-M1
BMC1-M4
Pat 35
BMC2
Pat 27
BMC3
Pat 38
b
Allele frequency
1
0.8
0.6
0.4
0.2
0
ZFHX3_p.G3249E
BRAF_p.V600E
FAT1_p.V3719M
MYC_p.N26S
RAC1_p.P29S
PLK2_p.P326L
PIK3CG_p.G829E
SETD2_p.Q2365E
INPP4A_p.S509N
PPM1D_p.R458X
SPTA1_p.E2148K
PTPRT_p.S73F
FLT1_p.L355F
PIK3CG_p.N522S
EPHA3_p.E338K
FLT1_p.E726K
PREX2_p.E213K
NOTCH1_p.T116I
TGFBR2_p.G251R
EPHA3_p.D848N
JUN_p.E282K
NRAS_p.Q61L
CDKN2A_p.P81L
YAP1_p.A268S
CARD11_p.D56N
GRIN2A_p.S1370F
BCORL1_p.S618N
MSH3_p.Y465C
PIK3C2G_p.E1160K
NRAS_p.G12C
CDC73_p.R91Q
BRAF_p.N581Y
SOCS1_p.Q210H
RET_p.G550E
RAD51B_p.K243R
NAB2_p.G221W
BRAF_p.V600K
PTEN_p.H296Y
SPTA1_p.A867E
NOTCH3_p.S1128P
MGA_p.L2573F
FGFR4_p.S431F
AR_p.Q72L
SMARCB1_p.D360H
BRD4_p.R113P
RBM10_p.A631P
MDC1_p.R1292Q
SMAD2_p.S458C
MAP3K1_p.E224X
JUN_p.G67E
EP300_p.M657T
BMC1-M4
Pat8/M1
BMC1-M1
Pat8/M3
Pat8/M4
Pat8/CSF
BMC3
Pat35
BMC2
Pat27
Pat38
c
Protein Acc
Q9UM47
Position
1128
AA1
S
AA2
P
Description
NOTCH3
PolyPhen2
This mutation is predicted to be probably damaging with a score of 0.997
(sensitivity: 0.41; specificity: 0.98) Confidence
HumDiv
0.00
0.20
0.40
0.60
0.80
1.00
This mutation is predicted to be probably damaging with a score of 0.986
(sensitivity: 0.54; specificity: 0.94) Confidence
HumVar
0.00
0.20
0.40
0.60
0.80
1.00
d
Allele frequency
1.0
0.8
0.6
0.4
0.2
0.0
M1
M3
M4
CSF
BMC1-M1
BMC1-M4
CARD11_p.D56N
BRAF_p.V600E
MYC_p.N26S
RAC1_p.P29S
NOTCH3_p.S1128P
MAG_p.L2573F

Supplementary fig. 7: TargetSeq of MBM-derived cell lines (BMCs) and concordant MBM provided insights into the genetic landscapes. a.) Oncoprint map depicts genetic modifications of 42 genes as determined by high-depth (>775x mean coverage) TargetSeq. Shown are missense (SNVs) and nonsense-mutations (STOP) and numbers of alterations per sample. b.) Representation of allele frequencies (color-coded) of mutations identified in (a). c.) PolyPhen2 prediction of a NOTCH3^{S1128P} mutation that was presented in a subclone of BMC1-M4 cells. d.) Changes of allele frequencies of indicated mutations among longitudinal metastases, CSF and cell lines of Pat8 demonstrating the permissive character of *BRAF*, *RAC1* and *MYC in vivo* and of *CARD11*, *NOTCH3* and MAG *in vitro*.

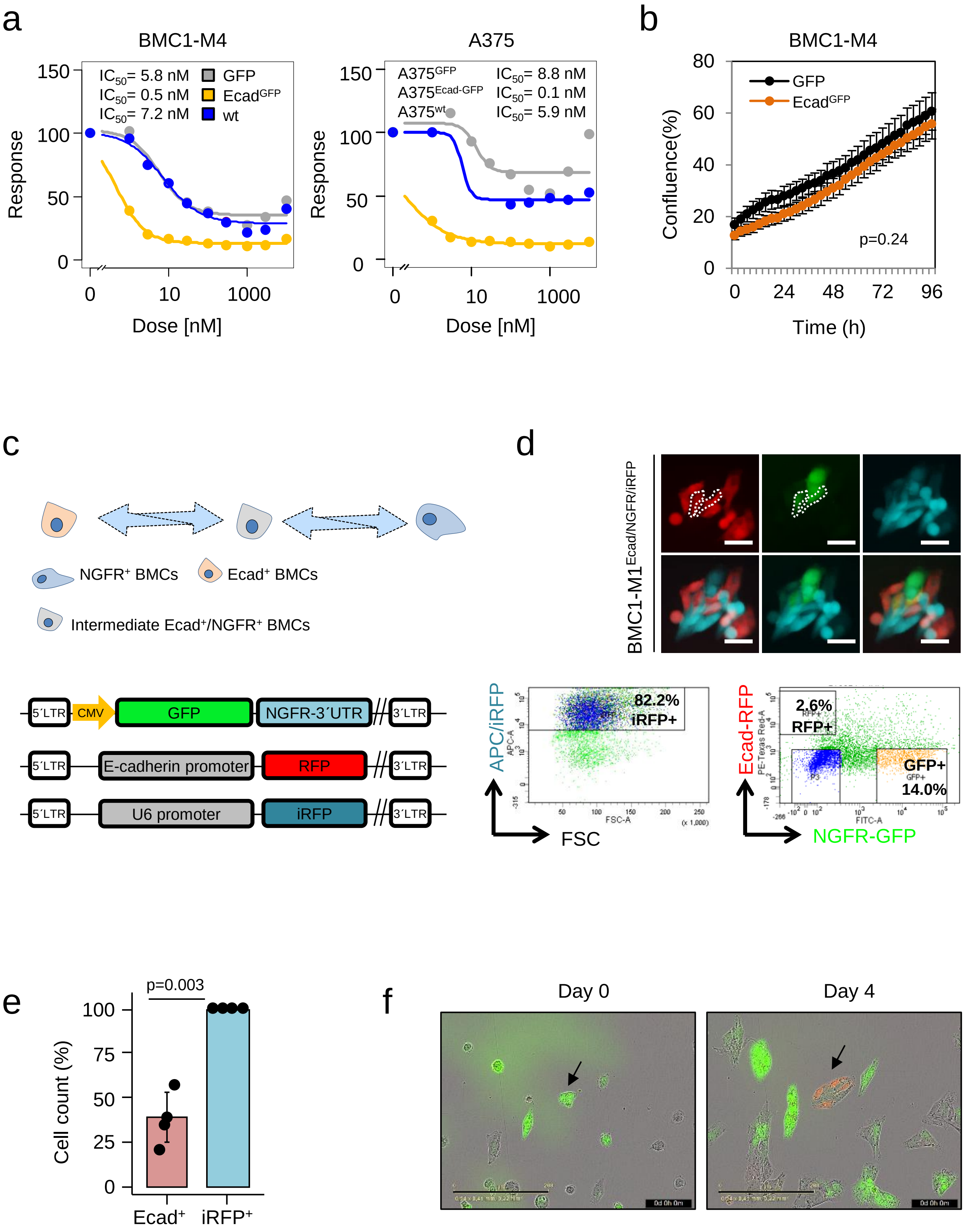

a
BMC1-M4
IC50= 5.8 nM GFP
IC50= 0.5 nM EcadGFP
IC50= 7.2 nM wt
Response
150
100
50
0
10
1000
Dose [nM]
A375
A375GFP IC50= 8.8 nM
A375Ecad-GFP IC50= 0.1 nM
A375wt IC50= 5.9 nM
b
BMC1-M4
GFP
EcadGFP
Confluence(%)
80
60
40
20
p=0.24
0
24
48
72
96
Time (h)
c
NGFR+ BMCs
Ecad+ BMCs
Intermediate Ecad+/NGFR+ BMCs
5´LTR
CMV
GFP
NGFR-3´UTR
3´LTR
E-cadherin promoter
RFP
U6 promoter
iRFP
d
BMC1-M1Ecad/NGFR/iRFP
APC/iRFP
82.2%
iRFP+
FSC
FSC-A
Ecad-RFP
2.6%
RFP+
GFP+
14.0%
NGFR-GFP
FITC-A
e
p=0.003
Cell count (%)
100
75
50
25
0
Ecad+
iRFP+
f
Day 0
Day 4

Supplementary fig. 8: A dual-reporter system enables the tracking of NGFR$^+$ or Ecad$^+$ subsets. a.) Dose-response of BMC1-M4 and A375 cells with expression of GFP or Ecad (Ecad^{GFP}) or non-transduced (wt) cells to dabrafenib. Calculated IC_{50} values demonstrate a sensitive phenotype of Ecad^{GFP} cells, not observed in wt or GFP expressing cells. b.) The live cell imaging-based tracking of BMC1-M4 cells expressing GFP or Ecad^{GFP} revealed that increased levels of Ecad not significantly ($p=0.24$) affected the proliferative capacity. Data are presented as mean values+/-SD of n=8 technical replicates depicting confluence (%). A representative of n=3 independent experiments is shown. c.) Upper panel: Scheme depicting a proposed interconnected relationship of Ecad$^+$, NGFR$^+$ and intermediate state BMCs. Lower panel: Linear plasmid maps of reporters enabling the indirect tracking of NGFR expression via a NGFR-specific 3´-UTR-sequence based regulation of GFP mRNA stability and expression or tracking of Ecad expression via Ecad-promoter controlled expression of RFP or general tracking of cells via constitutively expressed iRFP. d.) Upper panels: IF of reporter cells prior to sorting, depicting unique and co-expression of reporters. Bars indicate 50 µm. Lower panel: Fluorescence-activated cell sorting (FACS) based isolation of iRFP$^+$/RFP$^+$ BMC1-M1 cells. e.) Quantification of Ecad$^+$ cells 3d post FACS revealed a significant decreased level of Ecad$^+$ cells ($p=3.0e-03$). Values depict mean±SD of n=4 independent biological replicates. f.) Snapshots of NGFR$^+$ into Ecad$^+$ transitioning BMC1-M1 reporter cells (Movie 1) at days 0, 4. In (d) and (e) bars indicate 50 µm and 200 µm. In b, e significance was determined by a two-tailed unpaired t-test and paired t-test. Source data are provided as a Source Data file.

a

b

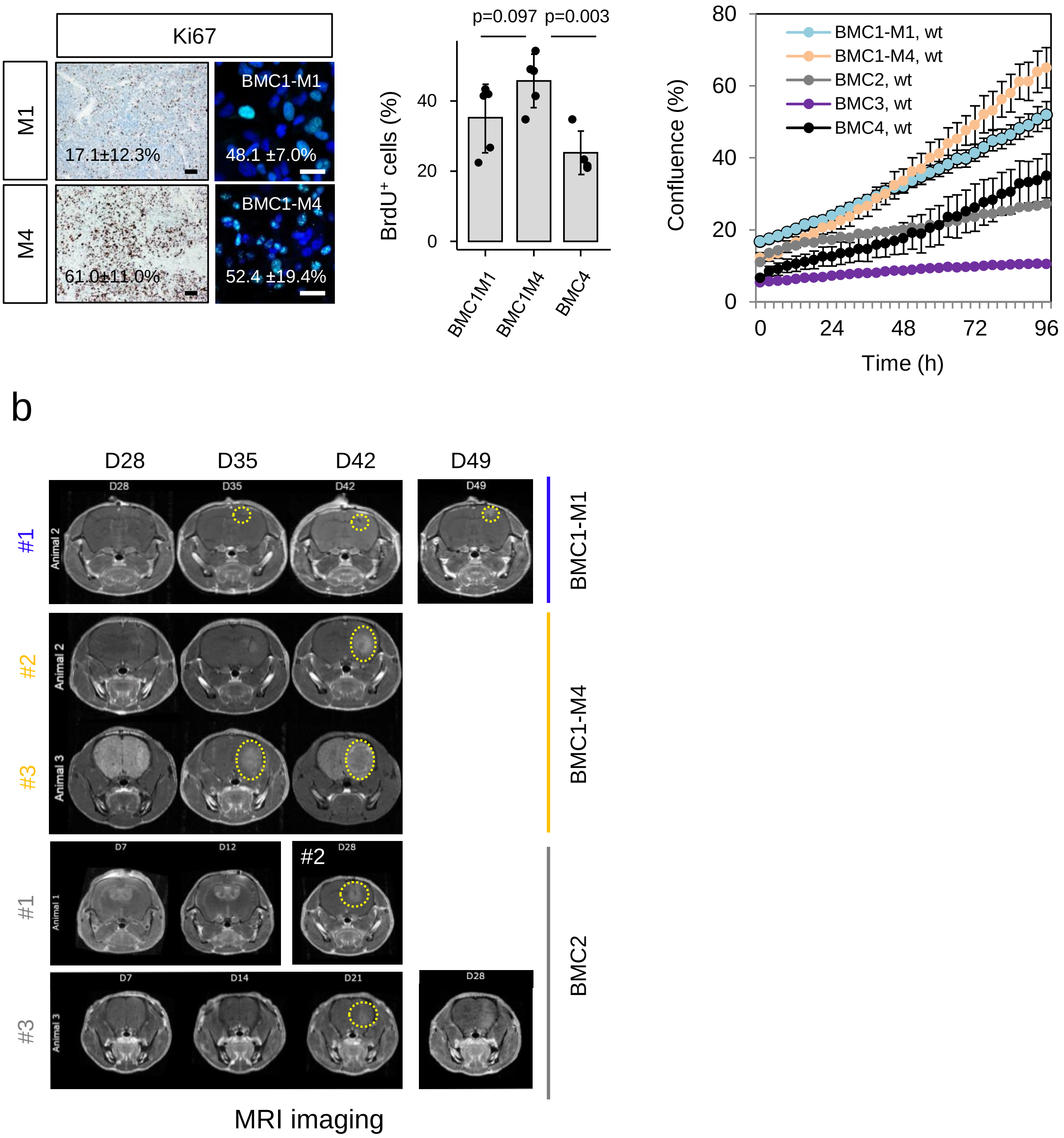

Ki67
M1
M4
17.1±12.3%
61.0±11.0%
BMC1-M1
BMC1-M4
48.1 ±7.0%
52.4 ±19.4%
p=0.097
p=0.003
BrdU+ cells (%)
BMC1M1
BMC1M4
BMC4
Confluence (%)
BMC1-M1, wt
BMC1-M4, wt
BMC2, wt
BMC3, wt
BMC4, wt
Time (h)
D28
D35
D42
D49
#1
#2
#3
BMC1-M1
BMC1-M4
BMC2
D7
D12
D14
D21
MRI imaging

Supplementary fig. 9: Assessment of the proliferative capacity of BMC. a.) Left panels: Content of proliferative, $Ki67^+$ cells in a drug-naïve (M1), therapy-resistant (M4) MBM and associated *in vitro* cell culture models. Center panel: Capacity of BrdU incorporation indicate the maintenance of a proliferative phenotype of M4-derived (BMC1-M4, p=0.003) cells *in vitro*. The level of $Ki67^+$ and $BrdU^+$ cells (% mean±SD) was determined by counting of stained cells of three independent experiments. Significance was determined by a two-tailed unpaired t-test. Source data are provided as a Source Data file. Right panel: Live-cell imaging based assessment of the proliferative capacity of indicated BMCs, revealed a separation into high (BMC1-M4, BMC2, BMC4) and low (BMC1-M1, BMC3) proliferative cells. b.) MRI images (T1) of contrasted animals were taken at indicated days (D). Dotted line depicts intracranial locations of tumors. Source data are provided as a Source Data file.

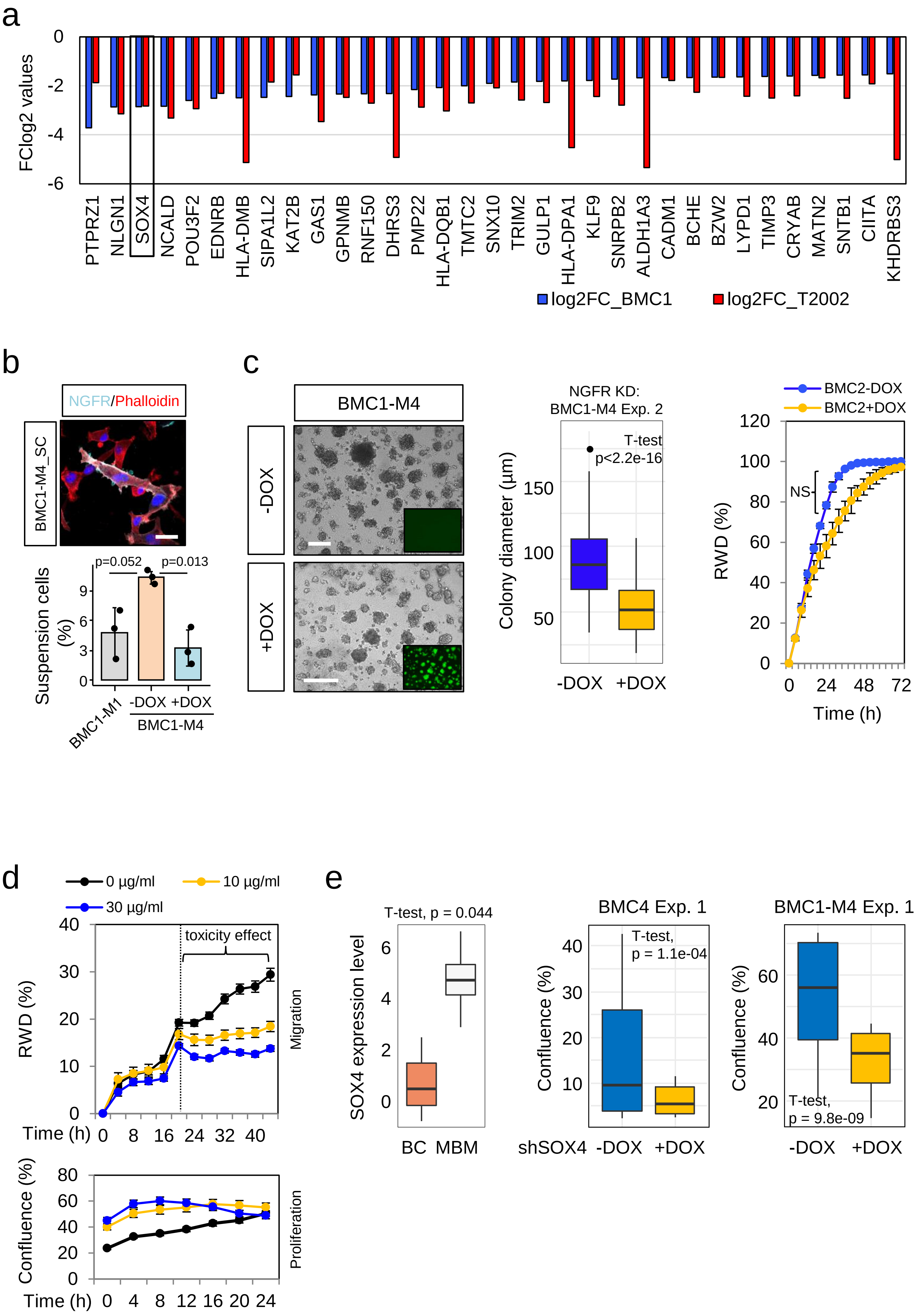
a
FClog2 values
0
-2
-4
-6
PTPRZ1
NLGN1
SOX4
NCALD
POU3F2
EDNRB
HLA-DMB
SIPA1L2
KAT2B
GAS1
GPNMB
RNF150
DHRS3
PMP22
HLA-DQB1
TMTC2
SNX10
TRIM2
GULP1
HLA-DPA1
KLF9
SNRPB2
ALDH1A3
CADM1
BCHE
BZW2
LYPD1
TIMP3
CRYAB
MATN2
SNTB1
CIITA
KHDRBS3
log2FC_BMC1
log2FC_T2002
b
NGFR/Phalloidin
BMC1-M4_SC
Suspension cells (%)
p=0.052
p=0.013
9
6
3
0
BMC1-M1
-DOX +DOX
BMC1-M4
c
BMC1-M4
-DOX
+DOX
NGFR KD:
BMC1-M4 Exp. 2
T-test
p<2.2e-16
Colony diameter (μm)
150
100
50
-DOX +DOX
BMC2-DOX
BMC2+DOX
RWD (%)
120
100
80
60
40
20
0
NS
0 24 48 72
Time (h)
d
0 μg/ml
10 μg/ml
30 μg/ml
toxicity effect
RWD (%)
40
30
20
10
0
Time (h) 0 8 16 24 32 40
Migration
Confluence (%)
80
60
40
20
0
Time (h) 0 4 8 12 16 20 24
Proliferation
e
T-test, p = 0.044
SOX4 expression level
6
4
2
0
BC MBM
BMC4 Exp. 1
T-test,
p = 1.1e-04
Confluence (%)
40
30
20
10
shSOX4 -DOX +DOX
BMC1-M4 Exp. 1
Confluence (%)
60
40
20
T-test,
p = 9.8e-09
-DOX +DOX

Supplementary fig. 9: Assessment of the proliferative capacity of BMC. a.) Left panels: Content of proliferative, Ki67$^+$ cells in a drug-naïve (M1), therapy-resistant (M4) MBM and associated *in vitro* cell culture models. Center panel: Capacity of BrdU incorporation indicate the maintenance of a proliferative phenotype of M4-derived (BMC1-M4, p=0.003) cells *in vitro*. The level of Ki67$^+$ and BrdU$^+$ cells (% mean±SD) was determined by counting of stained cells of three independent experiments. Significance was determined by a two-tailed unpaired t-test. Source data are provided as a Source Data file. Right panel: Live-cell imaging based assessment of the proliferative capacity of indicated BMCs, revealed a separation into high (BMC1-M4, BMC2, BMC4) and low (BMC1-M1, BMC3) proliferative cells. b.) MRI images (T1) of contrasted animals were taken at indicated days (D). Dotted line depicts intracranial locations of tumors. Source data are provided as a Source Data file.

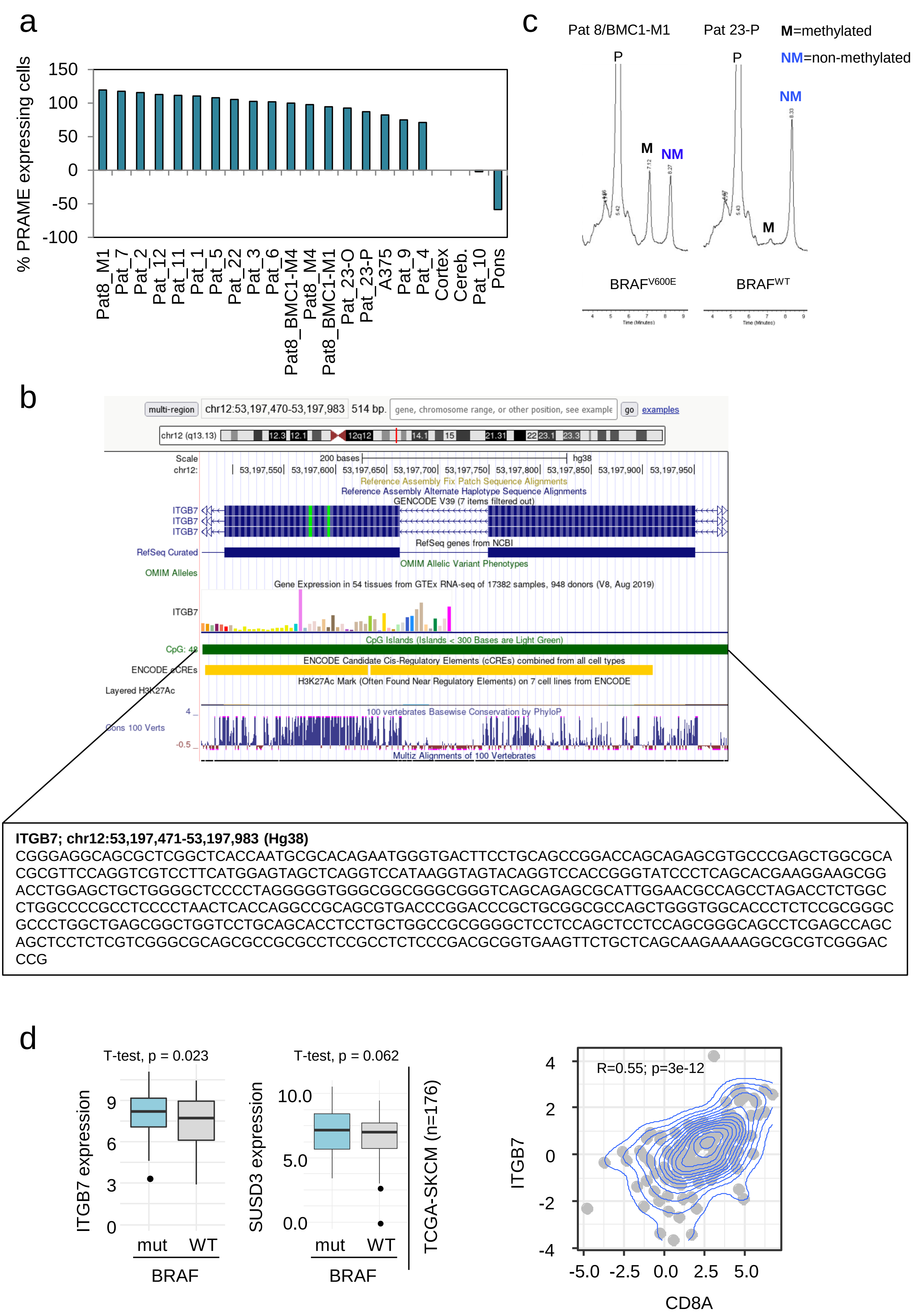
a
% PRAME expressing cells
150
100
50
0
-50
-100
Pat8_M1
Pat_7
Pat_2
Pat_12
Pat_11
Pat_1
Pat_5
Pat_22
Pat_3
Pat_6
Pat8_BMC1-M4
Pat8_M4
Pat8_BMC1-M1
Pat_23-O
Pat_23-P
A375
Pat_9
Pat_4
Cortex
Cereb.
Pat_10
Pons
c
Pat 8/BMC1-M1
Pat 23-P
M=methylated
NM=non-methylated
P
M
NM
BRAF V600E
BRAF WT
Time (Minutes)
b
multi-region
chr12:53,197,470-53,197,983 514 bp.
gene, chromosome range, or other position, see example
go
examples
chr12 (q13.13)
Scale
200 bases
hg38
chr12:
53,197,550 53,197,600 53,197,650 53,197,700 53,197,750 53,197,800 53,197,850 53,197,900 53,197,950
Reference Assembly Fix Patch Sequence Alignments
Reference Assembly Alternate Haplotype Sequence Alignments
GENCODE V39 (7 items filtered out)
ITGB7
ITGB7
ITGB7
RefSeq genes from NCBI
RefSeq Curated
OMIM Allelic Variant Phenotypes
OMIM Alleles
Gene Expression in 54 tissues from GTEx RNA-seq of 17382 samples, 948 donors (V8, Aug 2019)
ITGB7
CpG Islands (Islands < 300 Bases are Light Green)
CpG: 48
ENCODE Candidate Cis-Regulatory Elements (cCREs) combined from all cell types
ENCODE cCREs
H3K27Ac Mark (Often Found Near Regulatory Elements) on 7 cell lines from ENCODE
Layered H3K27Ac
100 vertebrates Basewise Conservation by PhyloP
4
Cons 100 Verts
-0.5
Multiz Alignments of 100 Vertebrates
ITGB7; chr12:53,197,471-53,197,983 (Hg38)
CGGGAGGCAGCGCTCGGCTCACCAATGCGCACAGAATGGGTGACTTCCTGCAGCCGGACCAGCAGAGCGTGCCCGAGCTGGCGCA
CGCGTTCCAGGTCGTCCTTCATGGAGTAGCTCAGGTCCATAAGGTAGTACAGGTCCACCGGGTATCCCTCAGCACGAAGGAAGCGG
ACCTGGAGCTGCTGGGGCTCCCCTAGGGGGTGGGCGGCGGGCGGGTCAGCAGAGCGCATTGGAACGCCAGCCTAGACCTCTGGC
CTGGCCCCGCCTCCCCTAACTCACCAGGCCGCAGCGTGACCCGGACCCGCTGCGGCGCCAGCTGGGTGGCACCCTCTCCGCGGGC
GCCCTGGCTGAGCGGCTGGTCCTGCAGCACCTCCTGCTGGCCGCGGGGCTCCTCCAGCTCCTCCAGCGGGCAGCCTCGAGCCAGC
AGCTCCTCTCGTCGGGCGCAGCGCCGCGCCTCCGCCTCTCCCGACGCGGTGAAGTTCTGCTCAGCAAGAAAAGGCGCGTCGGGAC
CCG
d
T-test, p = 0.023
ITGB7 expression
9
6
3
0
mut
WT
BRAF
T-test, p = 0.062
SUSD3 expression
10.0
5.0
0.0
mut
WT
BRAF
TCGA-SKCM (n=176)
R=0.55; p=3e-12
ITGB7
4
2
0
-2
-4
-5.0
-2.5
0.0
2.5
5.0
CD8A

Supplementary figure 11: Methylation defines molecular subsets in MBM a.) Determination of tumor cell content via expression of *PRAME* (%, related to BMC1-M1 cells. b.) Illustration of the CpG island within the ITGB7 gene as depicted by probe cg11510999 binding to region 53,591,490 on chromosome 12. c.) Representative chromatogram of a $BRAF^{V600}$ mutated and a $BRAF^{wt}$/$NRAS^{Q61}$ sample showing a different methylation index as determined by the specific formula (see Methods section). d.) Left and center panels: Box plots indicating a higher level of expression of *ITGB7* (p=0.023) but not SUSD3 in $BRAF^{mut}$ melanoma (EM only, n=176, TCGA-SKCM). Significance was determined by a unpaired two-tailed t-test. Right panel: Dot plot showing a significant (p=3e-12) correlation (R=0.55) of expression of *ITGB7* and of the marker of cytotoxic T cells *CD8A* of melanoma (primary, metastases; n=472 TCGA-SKCM melanoma). In (d) box and whisker plots show median (center line), the upper and lower quartiles (the box), and the range of the data (the whiskers), including outliers and significance was determined by a two-tailed unpaired t-test. Source data are provided as a Source Data file.
