# Supplementary material for: Decoding molecular programs in melanoma brain metastases
Source: Nat Commun. 2022 Nov 26;13:7304. doi: 10.1038/s41467-022-34899-x (PMC9701224; doi:10.1038/s41467-022-34899-x)
Supplement: Supplementary file 3 — Description of Additional Supplementary Files [file 41467_2022_34899_MOESM3_ESM.pdf]

Description of Additional Supplementary Files

Supplementary Data 1: Summary of patient´s MBM characteristics (Gender, BRAF/NRAS status of MBM, intracranial location and therapeutic interventions) and methods used for analysis.

Supplementary Data 2: Table containing genes specifying MBM (melanoma brain metastases; pan-MBM) or BC (brain controls) derived from the comparison of whole transcriptome data. Signature genes may enable the prediction of MBM purity.

Supplementary Data 3: Tables containing genes that are correlated and/or co-expressed in MBM with Ecad and NGFR.

Supplementary Data 4: Gene-set enrichment analysis (GSEA) of MBM regarding their phenotypes (Ecad, NGFR) or level of immune cell infiltration (TIL status).

Supplementary Data 5: Tables containing differential regulated genes among Pat8/M1 and concordant cell line BMC1-M1 providing insights into molecular changes occuring during the in vivo-to-in vitro transition of tumor cells.

Supplementary Data 6: Table providing information on TargetSeq parameters and about single nucleotide variants (SNVs) found by TargetSeq of MBM, n=7 and associated BMCs, n=4.

Supplementary Data 7: Table containing expression levels of progressive/core progressive genes of MBM, n=16 and BMCs, n=2.

Supplementary Data 8: Table providing information on NGFR-associated genes. Genes were found commonly downregulated in two different cell lines that stably expressed shRNAs targeting exon 3 (BMC1-M1) or exon 6 (T2002) of NGFR. Among the list of common NGFR-targets were progressive genes (n=6).

Supplementary Data 9: Tables containing sequences of qPCR primers.

Supplementary Data 10: Summary of recently identified (in this study) and previously published and gene signatures used for GSEA.

Supplementary Data 11: Table providing information on 46 differentially methylated regions (CpG islands in promoters, shelfs, shores as identified by the comparative methylome profiling of $BRAF^{V600E/K}$ and $BRAF^{wt}$ MBM.

Supplementary Movie 1: Ecad/NGFR-reporter cells enabled live tracking of cellular plasticity. Cellular plasticity, the non-genetic conversion of cellular phenotypes was traceable via a double (Ecad/NGFR) reporter system established in BMC1-M1 cells. Cells with loss of 3´-UTR-NGFR-GFP reporter activity spontaneously transitioned into Ecad (RFP)-reporter positive cells.

Supplementary Movie 2: Ecad/NGFR-reporter negative cells spontaneously acquired activation of the Ecad-promoter. The double reporter system enabled the tracking of spontaneous activation of the Ecad-promoter as monitored via expression of RFP.
