# Supplementary material for: Decoding molecular programs in melanoma brain metastases
Source: Nat Commun. 2022 Nov 26;13:7304. doi: 10.1038/s41467-022-34899-x (PMC9701224; doi:10.1038/s41467-022-34899-x)
Supplement: Supplementary file 17 — Reporting Summary [file 41467_2022_34899_MOESM17_ESM.pdf]

nature portfolio

Corresponding author(s): Josefine Radke, Torben Redmer

Last updated by author(s): Oct 30, 2022

Reporting Summary

Nature Portfolio wishes to improve the reproducibility of the work that we publish. This form provides structure for consistency and transparency in reporting. For further information on Nature Portfolio policies, see our Editorial Policies and the Editorial Policy Checklist.

Statistics

For all statistical analyses, confirm that the following items are present in the figure legend, table legend, main text, or Methods section.

n/a	Confirmed	
☐	☒	The exact sample size (*n*) for each experimental group/condition, given as a discrete number and unit of measurement
☐	☒	A statement on whether measurements were taken from distinct samples or whether the same sample was measured repeatedly
☐	☒	The statistical test(s) used AND whether they are one- or two-sided<br>*Only common tests should be described solely by name; describe more complex techniques in the Methods section.*
☐	☒	A description of all covariates tested
☐	☒	A description of any assumptions or corrections, such as tests of normality and adjustment for multiple comparisons
☐	☒	A full description of the statistical parameters including central tendency (e.g. means) or other basic estimates (e.g. regression coefficient) AND variation (e.g. standard deviation) or associated estimates of uncertainty (e.g. confidence intervals)
☐	☒	For null hypothesis testing, the test statistic (e.g. *F*, *t*, *r*) with confidence intervals, effect sizes, degrees of freedom and *P* value noted<br>*Give P values as exact values whenever suitable.*
☒	☐	For Bayesian analysis, information on the choice of priors and Markov chain Monte Carlo settings
☒	☐	For hierarchical and complex designs, identification of the appropriate level for tests and full reporting of outcomes
☐	☒	Estimates of effect sizes (e.g. Cohen's *d*, Pearson's *r*), indicating how they were calculated

Our web collection on statistics for biologists contains articles on many of the points above.

Software and code

Policy information about availability of computer code

Data collection	No software was used.

Data analysis	Gene set enrichment analysis was performed with GSEA standalone tool v4.1.0 [build:27].<br>Molecular signatures were used from molecular signature database MsigDB (Hallmark, C2) files v.7.4.<br>Single sample GSEA and generation of heat maps and box plots was performed using R and R-Studio Version 1.3.1093.<br>For RNA-sequencing analyses, Illumina bcl2fastq (2.19) was used for demultiplexing of sequenced reads and adapter trimming was performed with Skewer (version 0.2.2). The information on FASTQ files was obtained using the FastQC program (version 0.11.5-cegat) read out.<br>Raw sequencing data (fastq files) were quality controlled using fastqc (version 0.11.7 - Bioinformatics Group at the Babraham Institute, https://github.com/s-andrews/FastQC) and further preprocessed with fastp (https://github.com/OpenGene/fastp).<br>Reads were aligned to the GRCh38 version of the human genome using TopHat and counts per gene were calculated by the featureCount-algorithm from the Rsubread package. All further steps of the analysis were done in R (v.2022.07.1). Raw counts of protein-coding genes were normalized using the DESeq2 (https://bioconductor.org/packages/release/ bioc/html/DESeq2.html) package.<br>Targeted DNA-sequencing of cancer hotspot (CHP2v, ThermoFisher) or TruSight Oncology 500 panel (Illumina) libraries was performed with benchtop sequencers IonProton (Thermo fisher) or NextSeq2000 (Illumina).<br>Sequencing reads were mapped to human genome (v.GRCh38) and variants were called using variant caller implemented in Torrent Suite™ Software 5.14 or using Illumina/Pisces v.5.2.11 (https://github.com/Illumina/Pisces).<br>Disease-causing/probably damaging mutations were validated using COSMIC database, PolyPhen2 and Varsome integrated and plotted using ComplexHeatmap/oncoprint and R v3.4.0.<br>Methylome profiling: Raw data from Illumina Epic arrays (idat files) were preprocessed and analyzed in the standard workflow of the packages RnBeads (https://git.bioconductor.org/packages/RnBeads) and watermelon (https://git.bioconductor.org/packages/wateRmelon).<br>Images were edited with Adobe Photoshop 2020; Version: v.21.1.0.106x64

For manuscripts utilizing custom algorithms or software that are central to the research but not yet described in published literature, software must be made available to editors and reviewers. We strongly encourage code deposition in a community repository (e.g. GitHub). See the Nature Portfolio guidelines for submitting code & software for further information.

Data

Policy information about availability of data

All manuscripts must include a data availability statement. This statement should provide the following information, where applicable:

- Accession codes, unique identifiers, or web links for publicly available datasets
- A description of any restrictions on data availability
- For clinical datasets or third party data, please ensure that the statement adheres to our policy

Whole transcriptome and methylome data were deposited in the European Genome-Phenome Archive (EGA), under accession numbers EGAS00001005976 and EGAS00001005975 (https://ega-archive.org/). The data are available under controlled access. Access can be obtained by contacting the appropriate Data Access Committee listed in the study. Access will be granted to commercial and non-commercial parties according to patient consent forms and data transfer agreements. A response to requests for data access can be expected within 14 days. After access has been granted, the data is available for two years.
Supplementary tables have been deposited in Zenodo (https://zenodo.org/record/7013097). Kaplan-Meier survival data were derived from study EGAS00001003672 shown in Figure 8e and Supplementary fig. 4b are provided in the Source Data file with this paper.
Raw image files of histological stainings, immunofluorescence, and immunohistochemistry shown in the Figures have been deposited publicly at Zenodo https://doi.org/10.5281/zenodo.7249214]
Panel target sequencing results (SNVs) are given in Supplementary table 6.
SYBR Green quantitative real-time PCR (qPCR) results of SOX4, NGFR, AXL, MET, HERC5, TCF19 and FOSL1 iare given in Figure 7c (right).
Raw real-time PCR data (original xls file containing CT/RQ values) are given in the Source Data file with this paper.
Live cell imaging-based raw measurement files of drug response and proliferation assays are given in the Source Data file with this paper.
The remaining data are available within the article, Supplementary Information or Source Data file.

Field-specific reporting

Please select the one below that is the best fit for your research. If you are not sure, read the appropriate sections before making your selection.

☒ Life sciences ☐ Behavioural & social sciences ☐ Ecological, evolutionary & environmental sciences

For a reference copy of the document with all sections, see nature.com/documents/nr-reporting-summary-flat.pdf

Life sciences study design

All studies must disclose on these points even when the disclosure is negative.

Sample size	We investigated 48 melanoma brain metastases (MBM) of n=39 patients diagnosed with malignant melanoma and MBM-derived cell lines (n=5, BMCs). We performed whole transcriptome analysis of MBM specimens (n=16), BMCs (n=2) and normal brain tissue controls (n=3) as well as 850k methylome analysis of MBM (n=21). We performed Targeted deep-DNA sequencing of MBM (n=6) and BMCs (n=4) using the TruSight Oncology panel (comprised ~500 genes) and of MBM (n=24) and BMCs (n=3) using the Cancer HotSpot Panel v2 (comprised ~50 genes). No statistical methods were used to predetermine sample sizes.<br><br>We included all individuals (starting in 2017) with MBM where sufficient material was available as specified in the description of study design.<br><br>As whole transcriptome data of therapeutically (BRAFi, MEKi, ICi) treated MBM are not avaliable, we performed additional analyses on the following data sets: GSE77940 (pre- and post (BRAFi/MEKi) treatment melanoma (n= 6 patients; one was excluded from analysis). In addition

we performed analysis on the TCGA melanoma set (SKCM) containing 472 specimens (primary melanoma, n= 151; extracranial metastases, n=315 and brain metastases, n=6) and on expression data of FFPE samples of melanoma patiens (n=138) including intracranial (n= 79, BM) and extracranial metastases (n=59, EM) of study EGAS0000100367279 performed by Michael Davies.

Data exclusions	No data were excluded from analyses.
Replication	Histological diagnosis of melanoma samples was performed by at least two consultants of (neuro)pathology with agreement. Histological stainings were replicated at least once with the appropriate positive and negative controls. Each replication was successful. Immunohistochemistry/-fluorescent analyses were technically replicated at least once. Each replication was successful.<br><br>All cell-based in vitro experiments were performed in six to eight technical and three biological replicates. Each replication was successful. RT-qPCR was performed in two to three technical and three biological replicates. Each replication was successful. The reliability of TargetSeq was demonstrated by comparative analyses of concordant sets of MBM and/or associated sets of MBM and BMCs. In addition, overlapping analyses of two indendent amplicon-panels and prior results from routine diagnostic (at least BRAF status) successfully confirmed the reliability of DNA sequencing. Knockdown of NGFR was additionally validated by immunoblotting which was replicated in five cell lines. Each replication was successful.
Randomization	Randomization is not relevant for this study as we included all patients with MBM where sufficient material was available as described in the study design.
Blinding	Blinding was implemented through use of unsupervised analysis.<br>Targeted deep-DNA ssequencing, RNA-sequencing, real-time PCR, immunohistological stainings, IF stainings, and FACS analysis were performed in a blinded fashion.

Reporting for specific materials, systems and methods

We require information from authors about some types of materials, experimental systems and methods used in many studies. Here, indicate whether each material, system or method listed is relevant to your study. If you are not sure if a list item applies to your research, read the appropriate section before selecting a response.

Materials & experimental systems

n/a	Involved in the study	
	x	Antibodies
	x	Eukaryotic cell lines
x		Palaeontology and archaeology
	x	Animals and other organisms
	x	Human research participants
x		Clinical data
x		Dual use research of concern

Methods

n/a	Involved in the study	
x		ChIP-seq
	x	Flow cytometry
	x	MRI-based neuroimaging

Antibodies

Antibodies used

The following antibodies were used in our study:

Immunohistochemistry (IHC):
Rabbit Anti-Human p75NTR (company: Cell signaling, catalogue number: CST #8238, clone D4B3, mAb rabbit, LOT#:2, dilution: 1:100)
Rabbit Anti-Human CD3 (company: Agilent, catalogue number: #A045201-2, dilution: 1:100, LOT#: 20049827)
Mouse Anti-Human Ki67 (dilution: 1:100; company: DAKO; clone: Mib-1; catalogue: M7240; LOT#: 41315040)

For stainings with Rabbit Anti-Human p75NTR, Rabbit Anti-Human CD3, and Mouse Anti-Human Ki67 (listed above), we used an automated platform (VENTANA™). Primary antibody was applied and developed using the iVIEW DAB Detection Kit (Ventana Medical Systems; Catalog Number: 760-091).

Immunofluorescence (IF)
Rabbit Anti-Human p75NTR (company: Cell signaling, catalogue number: CST #8238, clone D4B3, LOT#:2, dilution: 1:100)
Rabbit Anti-Human E-cadherin (company: Cell signaling, catalogue number: CST #3195, clone 24E10, LOT#:13, dilution: 1:200)
Mouse Anti-Human E-cadherin (company: Santa Cruz, catalogue number: sc-8426, clone G10, LOT#:G0616, dilution: 1:50)
Rat Anti-Human E-cadherin-AlexaFluor647 (company: BioLegend, catalogue number: 147308, clone DECMA-1, LOT#:B268274, dilution: 1:50)
Mouse Anti-Human KBA.62 (company: NovusBiologicals, catalogue number: NBP2-45285, LOT#:MSM1-895P180523, dilution: 1:100)
Mouse Anti-Human GFAP-AlexaFluor594 (company: BioLegend, catalogue number: 644708, clone 2E1.E9, LOT#:B276277, dilution: 1:200)
Alexa Fluor® 488 Anti-BrdU (Isotype: Mouse IgG1, κ; compaby: BioLegend; catalogue number: 364105; clone: 3D4; dilution: 1:20; LOT#:B205040)
Mouse Anti-Human Ki67 (company: Cell signaling, catalogue number: CST #9449 clone 8D5, LOT#:4, dilution: 1:200)

FACS
Rat Anti-Human E-cadherin-AlexaFluor647 (company: BioLegend, catalogue number: 147308, clone DECMA-1, LOT#:B268274, dilution: 1:50)
Rabbit Anti-Human AXL (AXL (company: Atlas Antibodies, catalogue number: HPA037423, LOT#:A114302, dilution: 1:100)
Mouse Anti-Human-MET (company: Cell signaling, catalogue number: CST #8741; clone L6E7, LOT#:1, dilution: 1:100)
Alexa Fluor® 647 Anti-human CD271 (Isotype: Mouse IgG1, κ; company: BioLegend; catalogue number: 3345114; clone: ME20.4; dilution: 1:80; LOT#:B266105)
PE anti-human CD271 (Isotype: Mouse IgG1, κ, company: BioLegend; catalogue number: 3345106; clone: ME20.4; dilution: 1:80 LOT#:B262596)

Secondary Antibodies
Goat anti-Rabbit IgG (H+L) Highly Cross-Adsorbed Secondary Antibody, Alexa Fluor™ 555 (company: Invitrogen, catalogue number: #A21429; dilution: 1:500, LOT#:1010124)
Goat anti-Rabbit IgG (H+L) Cross-Adsorbed Secondary Antibody, Alexa Fluor™ 647 (company: Invitrogen, catalogue number: #A21245; dilution: 1:500, LOT#:1037285)
Goat anti-Rabbit IgG (H+L) Highly Cross-Adsorbed Secondary Antibody, Alexa Fluor™ 594 (company: Invitrogen, catalogue number: #A11037; dilution: 1:500, LOT#:1003217)
Goat anti-Rabbit IgG (H+L) Highly Cross-Adsorbed Secondary Antibody, Alexa Fluor™ 488 (company: Invitrogen, catalogue number: #A11034; dilution: 1:500, LOT#:1937195)
Goat anti-Mouse IgG (H+L) Highly Cross-Adsorbed Secondary Antibody, Alexa Fluor™ 647 (company: Invitrogen, catalogue number: #A21236; dilution: 1:500, LOT#:1003214)
Goat anti-Mouse IgG (H+L) Cross-Adsorbed Secondary Antibody, Alexa Fluor™ 488 (company: Invitrogen, catalogue number: #A11001; dilution: 1:500, LOT#:1907294)
Goat anti-Mouse IgG (H+L) Cross-Adsorbed Secondary Antibody, Alexa Fluor™ 594 (company: Invitrogen, catalogue number: #A11005; dilution: 1:500, LOT#:1890858)

Validation

All antibodies used in this study are validated for their application by the manufacturer and have been widely used in several studies (please see citations below). All antibodies were validated for the use of immunohistochemistry (IHC), FACS (FC) or Immunofluorescence (IF) by the manufacturer. Positive controls (tissue known to express the protein of interest) were either integrated as on-slide controls or as separate slide within the same (automated) IHC/IF run. The following positive (PC) and negative controls (NC) are used:

Rabbit Anti-Human p75NTR (company: Cell signaling, catalogue number: CST, #8238)
Applications (Manufacturer): IHC/IF/IB [Immunohistochemistry (IHC); Immunofluorescence (IF); Immunoblotting (IB); Flow cytometry/FACS (FC)]
(PC: tonsil (T-cell zone), human, murine and rat brains, human skin; NC: colon tissue, HEK293 cells, astroglia cells)
Citations: Wagoner MD, Bohrer LR, Aldrich BT, Greiner MA, Mullins RF, Worthington KS, Tucker BA, Wiley LA. Feeder-free differentiation of cells exhibiting characteristics of corneal endothelium from human induced pluripotent stem cells. Biol Open. 2018 May 8;7(5):bio032102. doi: 10.1242/bio.032102. PMID: 29685994; PMCID: PMC5992532.

Rabbit Anti-Human E-cadherin (company: Cell signaling, catalogue number: CST#3195)
Applications (Manufacturer): IHC/IF/IB [Immunohistochemistry (IHC); Immunofluorescence (IF); Immunoblotting (IB); Flow cytometry/FACS (FC)]
PC: WM35 cells (non-metastatic E-cadherin expressing melanoma cell line), human and dog colon tissue, murine embryonic stem cells; NC: murine and human fibroblasts)
Citation: Russell JP, Lim X, Santambrogio A, Yianni V, Kemkem Y, Wang B, Fish M, Haston S, Grabek A, Hallang S, Lodge EJ, Patist AL, Schedl A, Mollard P, Nusse R, Andoniadou CL. Pituitary stem cells produce paracrine WNT signals to control the expansion of their descendant progenitor cells. Elife. 2021 Jan 5;10:e59142. doi: 10.7554/eLife.59142. PMID: 33399538; PMCID: PMC7803373.

Mouse Anti-Human E-cadherin (company: Santa Cruz, catalogue number: sc-8426)
Applications (Manufacturer): WB, IP, IF, IHC(P) , ELISA
(PC: WM35 cells (non-metastatic E-cadherin expressing melanoma cell line), human and dog colon tissue, murine embryonic stem cells; NC: murine and human fibroblasts)
Citation: He Y, Hua R, Yang Y, Li B, Guo X, Li Z. LncRNA JPX Promotes Esophageal Squamous Cell Carcinoma Progression by Targeting miR-516b-5p/VEGFA Axis. Cancers (Basel). 2022 May 31;14(11):2713. doi: 10.3390/cancers14112713. PMID: 35681693; PMCID: PMC9179376.

Rat Anti-Human E-cadherin-AlexaFluor647 (company: BioLegend, catalogue number: 147308)
Applications (Manufacturer): FC, ICC, IHC-F, 3D IHC
(PC: WM35 cells (non-metastatic E-cadherin expressing melanoma cell line), human and dog colon tissue, murine embryonic stem cells; NC: murine and human fibroblasts)
Citation: Radtke AJ, Chu CJ, Yaniv Z, Yao L, Marr J, Beuschel RT, Ichise H, Gola A, Kabat J, Lowekamp B, Speranza E, Croteau J, Thakur N, Jonigk D, Davis JL, Hernandez JM, Germain RN. IBEX: an iterative immunolabeling and chemical bleaching method for high-content imaging of diverse tissues. Nat Protoc. 2022 Feb;17(2):378-401. doi: 10.1038/s41596-021-00644-9. Epub 2022 Jan 12. PMID: 35022622.

Mouse Anti-Human KBA.62 (company: NovusBiologicals, catalogue number: NBP2-45285,
Application (Manufacturer): ICC/IF, IHC, IHC-P, ICC/IF
(PC: WM35 cells (non-metastatic E-cadherin expressing melanoma cell line), NC: human brain tissue, tonsil)

Mouse Anti-Human GFAP-AlexaFluor594 (company: BioLegend, catalogue number: 644708)
Application (Manufacturer): IHC-F
(PC: human brain tissue, cortex, cerebellum; NC: colon tissue)
Citation: Watson AM, Rose AH, Gibson GA, Gardner CL, Sun C, Reed DS, Lam LKM, St Croix CM, Strick PL, Klimstra WB, Watkins SC. Ribbon scanning confocal for high-speed high-resolution volume imaging of brain. PLoS One. 2017 Jul 7;12(7):e0180486. doi: 10.1371/journal.pone.0180486. PMID: 28686653; PMCID: PMC5501561.

Rabbit Anti-Human AXL (company: Cell signaling, catalogue number: CST, #8661)
Application (Manufacturer): IHC/IF/IB [Immunohistochemistry (IHC); Immunofluorescence (IF); Immunoblotting (IB); Flow cytometry/ FACS (FC)]
(PC: Glioblastoma specimens, NC: tonsil (T-cell zone))
Citation: McNeal AS, Belote RL, Zeng H, Urquijo M, Barker K, Torres R, Curtin M, Shain AH, Andtbacka RH, Holmen S, Lum DH, McCalmont TH, VanBrocklin MW, Grossman D, Wei ML, Lang UE, Judson-Torres RL. BRAFV600E induces reversible mitotic arrest in human melanocytes via microrna-mediated suppression of AURKB. Elife. 2021 Nov 23;10:e70385. doi: 10.7554/eLife.70385. PMID: 34812139; PMCID: PMC8610417.

Mouse Anti-Human Ki67 (company: Cell signaling)
Application: ICC/IF, IHC, IHC-P, FC
(PC: tonsil (germinal center); NC: tonsil (T-cell zone))
Citation: Pascoal S, Salzer B, Scheuringer E, Wenninger-Weinzierl A, Sturtzel C, Holter W, Taschner-Mandl S, Lehner M, Distel M. A Preclinical Embryonic Zebrafish Xenograft Model to Investigate CAR T Cells In Vivo. Cancers (Basel). 2020 Feb 29;12(3):567. doi: 10.3390/cancers12030567. PMID: 32121414; PMCID: PMC7139560.

BrdU anti-BrdU-AlexaFluor488 (company: BioLegend)
Application: IF
Citation: Kaymak A, Sayols S, Papadopoulou T, Richly H. Role for the transcriptional activator ZRF1 in early metastatic events in breast cancer progression and endocrine resistance. Oncotarget. 2018 Jun 19;9(47):28666-28690. doi: 10.18632/oncotarget.25596. PMID: 29983888; PMCID: PMC6033359.

Rabbit Anti-Human AXL (AXL (company: Atlas Antibidies)
Application: IHC
Citation: Liu CA, Harn HJ, Chen KP, Lee JH, Lin SZ, Chiu TL. Targeting the Axl and mTOR Pathway Synergizes Immunotherapy and Chemotherapy to Butylidenephthalide in a Recurrent GBM. J Oncol. 2022 May 18;2022:3236058. doi: 10.1155/2022/3236058. PMID: 35646111; PMCID: PMC9132698.

Mouse Anti-Human-MET (company: Cell signaling)
Application: IF, FC
Citation: Kong LR, Mohamed Salleh NAB, Ong RW, Tan TZ, Syn NL, Goh RM, Fhu CW, Tan DSW, Iyer NG, Kannan S, Verma CS, Lim YC, Soo R, Ho J, Huang Y, Lim JSJ, Yan BJ, Nga ME, Lim SG, Koeffler HP, Lee SC, Kappei D, Hung HT, Goh BC. A common MET polymorphism harnesses HER2 signaling to drive aggressive squamous cell carcinoma. Nat Commun. 2020 Mar 25;11(1):1556. doi: 10.1038/ s41467-020-15318-5. PMID: 32214092; PMCID: PMC7096530.

Alexa Fluor® 647 Anti-human CD271 (company: BioLegend)
Application: FC
Citation: Naamati A, Williamson JC, Greenwood EJ, Marelli S, Lehner PJ, Matheson NJ. Functional proteomic atlas of HIV infection in primary human CD4+ T cells. Elife. 2019 Mar 12;8:e41431. doi: 10.7554/eLife.41431. PMID: 30857592; PMCID: PMC6414203

PE anti-human CD271 (company: BioLegend)
Application: FC
Citation: Asada S, Goyama S, Inoue D, Shikata S, Takeda R, Fukushima T, Yonezawa T, Fujino T, Hayashi Y, Kawabata KC, Fukuyama T, Tanaka Y, Yokoyama A, Yamazaki S, Kozuka-Hata H, Oyama M, Kojima S, Kawazu M, Mano H, Kitamura T. Mutant ASXL1 cooperates with BAP1 to promote myeloid leukaemogenesis. Nat Commun. 2018 Jul 16;9(1):2733. doi: 10.1038/s41467-018-05085-9. PMID: 30013160; PMCID: PMC6048047.

Rabbit Anti-Human CD3 (company: Agilent)
(PC: tonsil (germinal center); NC: tonsil (T-cell zone))
Application: IHC
Citation: Bouafia A, Lofek S, Bruneau J, Chentout L, Lamrini H, Trinquand A, Deau MC, Heurtier L, Meignin V, Picard C, Macintyre E, Alibeu O, Bras M, Molina TJ, Cavazzana M, André-Schmutz I, Durandy A, Fischer A, Oksenhendler E, Kracker S. Loss of ARHGEF1 causes a human primary antibody deficiency. J Clin Invest. 2019 Mar 1;129(3):1047-1060. doi: 10.1172/JCI120572. Epub 2019 Feb 4. PMID: 30521495; PMCID: PMC6391114.

Ki67 (PC: tonsil (germinal center); NC: tonsil (T-cell zone))
References for Ki67:
Zhu X, Chen L, Huang B, Wang Y, Ji L, Wu J, et al. The prognostic and predictive potential of Ki-67 in triple-negative breast cancer. Sci Rep. 2020;10:225
Oyama Y, Nishida H, Kusaba T, Kadowaki H, Arakane M, Okamoto K, et al. Colon adenoma and adenocarcinoma with clear cell components - two case reports. Diagn Pathol. 2019;14:37

Standardized validation proceedure for all antibodies used in this study:

All antibodies were checked for reproducability and integrity of the assay three times in independent staining experiments and in at least three different positive samples and compared to expected staining patterns in the controls regarding published expression of the antigens were applicable. Furthermore an additional secondary-only antibody control (i.e. omission of first antibody) was performed for every setup.

Eukaryotic cell lines

Policy information about cell lines

Cell line source(s)	A375 (CRL-1619), WM35 (CRL-2807, Discontinued), and MeWo cells were purchased from ATCC. BMCs were established from intraoperative MBM (Patient-derived). Patient-derived T2002 cells were established from a intraoperative ymph-node metastases of a melanoma patient in a prievious project (Citation: Redmer T, Welte Y, Behrens D, Fichtner I, Przybilla D, Wruck W, Yaspo ML, Lehrach H, Schäfer R, Regenbrecht CR. The nerve growth factor receptor CD271 is crucial to maintain tumorigenicity and stem-like properties of melanoma cells. PLoS One. 2014 May 5;9(5):e92596. doi: 10.1371/journal.pone.0092596. Erratum in: PLoS One. 2014;9(8):e105274. PMID: 24799129; PMCID: PMC4010406.)
Authentication	Patient-derived cell lines have been authenticated by TargetSeq using the Ion AmpliSeq Sample ID human SNP genotyping panel and validated the concordance with tumors.
Mycoplasma contamination	Mycoplasma testing is routinely performed via DAPI staining. Addionally, the BMC cell lines were tested using a PCR Mycoplasma Test Kit (PCR Mycoplasma Test Kit I/C, catalogue number: PK-CA91-1024, company: PromoCell bioscience) All cell lines were free of mycoplasma at the time when the assays were performed.
Commonly misidentified lines (See ICLAC register)	None

Animals and other organisms

Policy information about studies involving animals; ARRIVE guidelines recommended for reporting animal research

Laboratory animals	Female CD-1 nude mice (8-9 weeks of age, 24-26g, Charles River Laboratories) were used for intrcranial injection experiments. The animals were maintained in a 12-hour light-dark cycle, at temperatures of 22°C (+/- 2°C) with 55% (+/-10%) humidity, and had ad libitum access to water and food.
Wild animals	No wild animals were used in this study
Field-collected samples	No field-collected samples were used in this study
Ethics oversight	All experiments with animals were performed in accordance with the German Animal Protection Law under the permission number G0130/20 obtained via the Berlin Ministry of Health and Social Affairs (LaGeSo). ARRIVE 2.0 Guidelines were strictly followed.

Note that full information on the approval of the study protocol must also be provided in the manuscript.

Human research participants

Policy information about studies involving human research participants

Population characteristics	Intraoperative, snap frozen or paraffin embedded brain metastases and extracranial metastases of patients (age: ≥18) diagnosed with melanoma and treated and operated at the Department of Neurosurgery, Charité-Universitätsmedizin Berlin, Berlin Germany. The available population characteristics are given in Supplementary table 1. Our cohort comprized 21 female and 19 male patients giving a female:male ratio of 1: 0.9
Recruitment	Not applicable. We enrolled all adult patients with melanoma brain metastases where sufficient material was available.
Ethics oversight	All patients gave written informed consent for the collection and scientific use of tumor material which was collected at the Biobank of the Charité – Comprehensive Cancer Center (CCCC) following ethics approval by the Ethics Committee of the Charité (EA1/152/10; EA1/107/17; EA4/028/18). In addition, thirty-two MBM archived at the Department of Neuropathology, Charité-Universitätsmedizin Berlin, Germany, were included in the study and analyzed. The usage of archived (FFPE) melanoma and central nervous system-derived control samples (pons, cortex, cerebellum) has been reviewed and approved by the Ethics Committee of the Charité (EA1/107/17 and EA1/075/19) and was in compliance with the Declaration of Helsinki.

Note that full information on the approval of the study protocol must also be provided in the manuscript.

Flow Cytometry

Plots

Confirm that:

- [x] The axis labels state the marker and fluorochrome used (e.g. CD4-FITC).
- [x] The axis scales are clearly visible. Include numbers along axes only for bottom left plot of group (a 'group' is an analysis of identical markers).
- [x] All plots are contour plots with outliers or pseudocolor plots.
- [x] A numerical value for number of cells or percentage (with statistics) is provided.

Methodology

Sample preparation	After removal of medium, cells were washed with PBS and harvested by Trypsin (0.05 % 206 Trypsin/EDTA). Following addition of cell culture medium, cells were collected by centrifugation at 330g at room temperature for 3 (min) and resuspended in 100 µl of ice cold buffer (PBS/0.5 % bovine serum/2 mM EDTA) and stored on ice. Cells were incubated with fluorescently labeled primary antibodies against CD271-PE (Miltenyi) DECMA1-APC (recognizing the N-terminal domain of E-cadherin, Biolegend), and non-labeled antibodies against AXL (Novus biologicals), PD-L1 (BioLegend), c-MET (MET, Cell signaling) or KBA.62 (BioLegend) diluted in buffer according to the manufacturer's specifications and stored at 4°C for 10 min to achieve proper labeling. Following, cells were washed by addition of buffer, collected by centrifugation and resuspended in 100µl of buffer that contained secondary 215 antibodies (AlexaFluor-488/594/647) and/or DAPI, diluted according to the manufacturer's specifications. After incubation for 10 min at 4°C and washing, cells were resuspended in 500 µl PBS and analyzed by flow cytometry (Canto II) or fractioned by FACS using a FACSAria™III cell sorter (Becton&Dickinson, BD). FACS-isolated cells were collected in cell culture medium and seeded on appropriate vessels following centrifugation. Data analysis was performed with FlowJo (Ver 10.7.1).
Instrument	Flow cytometric analyses were performed with FACS Canto II and sorting was performed using a FACS Aria III
Software	Data analysis was performed with FlowJo (Ver 10.7.1).
Cell population abundance	Reporter cells carrying NGFR and Ecad reporters were enriched
Gating strategy	The population was defined by Forward (FSC)- and Side-Scatter (SSC) characteristics, excluding low sized cell debris and apoptotic cells (DAPI positive cells). Following, unstained control cells were investigated for adjustment of laser power and chanels for subsequenc analysis of stained cells. A figure exemplifying the gating strategy is provided in Supplementary figure 6d.

- [x] Tick this box to confirm that a figure exemplifying the gating strategy is provided in the Supplementary Information.

Magnetic resonance imaging

Experimental design

Design type	Female CD-1 nude mice (8-9 weeks of age, 24-26g, Charles River Laboratories) were stereotactically inoculated with 2.5E+04 BMC1-M1 and BMC1-M4 cells using a 1µl Hamilton syringe and a stereotactic frame as described previously. Briefly, the bur hole was placed 2 mm lateral (right) and 1 mm rostral from the bregma. The cells were administered at a depth of 3 mm. The number of cells used for the inoculation was determined in accordance with previous literature with the established human melanoma cell line M14.
Design specifications	For the procedure, the animals received anesthesia (9mg Ketamine-Hydrochloride (CP-Pharma Handelsgesellschaft mbH, Burgdorf, Germany) + 1mg Xylazine (CP-Pharma Handelsgesellschaft mbH, Burgdorf, Germany) per 100g) intraperitoneally as well as subcutaneous prophylaxis against infection (10'000 I.E, benzylpenicillin potassium, InfectoPharm Arzneimittel und Consilium GmbH, Heppenheim, Germany) and analgesia (100 mg/kg Paracetamol (B. Braun Deutschland GmbH & Co. KG, Melsungen, Germany), Lidocaine (Aspen Germany GmbH, Munich, Germany). Additionally, analgesia (300 mg/kg*d Paracetamol, bene-Arzneimittel GmbH, Munich, Germany) was administered via the drinking water for the first two postoperative days. MRI scans were performed every 7 days until either the tumor volume was above 20 mm^3 or at latest on the 49th day after implantation.
Behavioral performance measures	Not applicable.

Acquisition

Imaging type(s)	NA
Field strength	7 Tesla
Sequence & imaging parameters	T1 weighted sequences (TR = 1,000 ms, TE = 10 416 ms, RARE factor = 2, 3 averages for BioSpec; TR = 975 ms, TE = 11.5 ms, RARE factor = 2, 4 averages for PharmaScan) after intraperitoneal administration of gadolinium-based contrast agent (12,09 mg per mouse in a solution with 180 µl 0,9% NaCl, Gadovist, Bayer AG, Leverkusen) and T2 (TR = 4,200 ms, TE = 36 ms, RARE factor = 8, 3 averages for Biospec and TR = 4,200 ms, TE = 36 ms, RARE factor = 8, 4 averages for

PharmaScan) were measured.

Area of acquisition: Whole brain scan

Diffusion MRI ☐ Used ☒ Not used

Preprocessing

Preprocessing software	ParaVision 6.0.1 or 5.1 412 software
Normalization	Not applicable
Normalization template	Not applicable
Noise and artifact removal	During the scans, the mice received inhalation anesthesia (1.0-1.5% Isoflurane (CP-Pharma Handelsgesellschaft mbH, Burgdorf, Germany) in a mixture of 30% oxygen and 70% nitrous oxide). The depth of the anesthesia was monitored using the respiratory frequency (70-120 breaths per minute).
Volume censoring	The tumor volume was measured using ITK-SNAP 3.8.0 Software (Paul A. Yushevich, Guido Gerig, www.itksnap.org).

Statistical modeling & inference

Model type and settings	Tumor volume of BMC1-M1 or BMC1-M4 inoculated mice were determined and indicated as mean of n=3 animals per group. As statistical testing was not performed.
Effect(s) tested	*Define precise effect in terms of the task or stimulus conditions instead of psychological concepts and indicate whether ANOVA or factorial designs were used.*

Specify type of analysis: ☒ Whole brain ☐ ROI-based ☐ Both

Statistic type for inference (See Eklund et al. 2016)	*Specify voxel-wise or cluster-wise and report all relevant parameters for cluster-wise methods.*
Correction	*Describe the type of correction and how it is obtained for multiple comparisons (e.g. FWE, FDR, permutation or Monte Carlo).*

Models & analysis

n/a	Involved in the study
☐	☐ Functional and/or effective connectivity
☐	☒ Graph analysis
☐	☐ Multivariate modeling or predictive analysis

Functional and/or effective connectivity	Not applicable
Graph analysis	Tumore volume relativ to time points of observation/MRI scans was specified by curve charts, using GraphPad Prism.
Multivariate modeling and predictive analysis	Not applicable
